# Whole genome sequencing–based analysis of tuberculosis (TB) in migrants: rapid tools for cross-border surveillance and to distinguish between recent transmission in the host country and new importations

**DOI:** 10.2807/1560-7917.ES.2019.24.4.1800005

**Published:** 2019-01-24

**Authors:** Estefanía Abascal, Laura Pérez-Lago, Miguel Martínez-Lirola, Álvaro Chiner-Oms, Marta Herranz, Imane Chaoui, Iñaki Comas, My Driss El Messaoudi, José Antonio Garrido Cárdenas, Sheila Santantón, Emilio Bouza, Darío García-de-Viedma

**Affiliations:** 1 Servicio Microbiología Clínica y Enfermedades Infecciosas, Hospital General Universitario Gregorio Marañón, Madrid, Spain; 2 Instituto de Investigación Sanitaria Gregorio Marañón, Madrid, Spain; 3 These authors have contributed equally; 4 Complejo Hospitalario Torrecárdenas, Almería, Spain; 5 Unidad Mixta Genómica y Salud, Centro Superior de Investigación en Salud Pública (FISABIO)-Universitat de València, Valencia, Spain; 6 CIBER Enfermedades respiratorias (CIBERES), Spain; 7 Unité de Biologie et Recherches Médicales, Division des Sciences du Vivant, Centre National de l'Energie, des Sciences et des Techniques Nucléaires (CNESTEN), Rabat, Morocco; 8 Instituto de Biomedicina de Valencia (IBV) Consejo Superior de Investigaciones Científicas (CSIC), Valencia, Spain; 9 CIBER Epidemiología y Salud Pública (CIBERESP), Spain; 10 Institut Pasteur of Morocco, Casablanca, Morocco; 11 Servicio de Secuenciación, Universidad de Almería, Almería, Spain; 12 Departamento de Medicina, Facultad de Medicina, Universidad Complutense de Madrid, Madrid, Spain

**Keywords:** tuberculosis, TB, molecular epidemiology, immigration, transmission, importation, whole genome sequencing, WGS, surveillance, cross-border surveillance, migrants

## Abstract

**Background:**

The analysis of transmission of tuberculosis (TB) is challenging in areas with a large migrant population. Standard genotyping may fail to differentiate transmission within the host country from new importations, which is key from an epidemiological perspective.

**Aim:**

To propose a new strategy to simplify and optimise cross-border surveillance of tuberculosis and to distinguish between recent transmission in the host country and new importations

**Methods:**

We selected 10 clusters, defined by 24-locus mycobacterial interspersed repetitive unit-variable number tandem repeat (MIRU-VNTR), from a population in Spain rich in migrants from eastern Europe, north Africa and west Africa and reanalysed 66 isolates by whole-genome sequencing (WGS). A multiplex-allele-specific PCR was designed to target strain-specific marker single nucleotide polymorphisms (SNPs), identified from WGS data, to optimise the surveillance of the most complex cluster.

**Results:**

In five of 10 clusters not all isolates showed the short genetic distances expected for recent transmission and revealed a higher number of SNPs, thus suggesting independent importations of prevalent strains in the country of origin. In the most complex cluster, rich in Moroccan cases, a multiplex allele-specific oligonucleotide-PCR (ASO-PCR) targeting the marker SNPs for the transmission subcluster enabled us to prospectively identify new secondary cases. The ASO-PCR-based strategy was transferred and applied in Morocco, demonstrating that the strain was prevalent in the country.

**Conclusion:**

We provide a new model for optimising the analysis of cross-border surveillance of TB transmission in the scenario of global migration.

## Background

International migration has modified the epidemiology of tuberculosis (TB) in most high-income countries and today, migrants account for up to 40–60% of cases in large cities [1-4]. Some cases are reactivations of infections acquired in the country of origin, with the remainder resulting from recent transmission after arrival in the host country.

Molecular epidemiology provides more accurate data on the transmission dynamics of TB in settings with a complex composition of cases due to migration [5-7]. Several studies have shown variable composition in the nationalities comprising transmission clusters. This variety ranges from settings with marked transmission permeability leading to multinational clusters, to other socio-epidemiological contexts where a more homogeneous composition of nationalities is found, with clusters only involving single nationalities [6,8]. Autochthonous clusters and those comprising several nationalities more likely reflect recent transmission events. However, clusters rich in cases from one country of origin are especially difficult to interpret. This is because they can be the result of either of two circumstances: (i) a strain is imported from the country of origin and subsequently transmitted to migrants of the same nationality in the host country; or (ii) genetically closely related strains, which are prevalent in the country of origin, are independently imported by individuals who were exposed in the country of origin but are not epidemiologically related in the host country. Thus, differentiation between these alternatives, i.e. recent transmission in the host country vs importation, is challenging, yet highly relevant in epidemiological terms.

Application of whole-genome sequencing (WGS) for analysis of transmission of TB has given birth to the field of genomic epidemiology, which has markedly increased specificity in the definition of transmission clusters [9-12]. Determination of the number of single nucleotide polymorphisms (SNPs) [12] between the sequences of different isolates allows to split clusters that had been previously defined by standard molecular tools into smaller subclusters that are much more consistent with the geographic distribution of the cases and with the epidemiological links between them [11].

Our aim was to apply WGS in a more in-depth analysis of migrant TB cases involved in clusters in Spain that had been defined by standard genotyping. We attempted to determine whether the clusters corresponded to recent transmission in the host country (because *Mycobacterium tuberculosis* (MTB) isolates show no or a very short genetic distance) or to undetected independent importations of strains that are prevalent in the country of origin and have acquired higher SNP-based diversity as a result of prolonged periods of circulation. In addition, we took advantage of the SNPs identified for either the recently transmitted or imported isolates, to tailor simple PCR tools to simplify and optimise the precise assignation of recent transmission or importation in the new clusters arising. Further, we used these same tools in a new extended and cross-border analysis, for an in-depth surveillance of the MTB strains analysed in unrelated Spanish populations, as well as in the country of origin.

## Methods

### Clusters and strains selected

We retrospectively selected all clusters from the ongoing molecular epidemiology universal genotyping programme in Almería, south-east Spain [7,13] fulfilling the following selection criteria: The clusters analysed were 24 locus mycobacterial interspersed repetitive unit-variable number tandem repeat (MIRU-VNTR)-defined clusters [14] including four or more cases, covering at least 5 years and rich (>60% of the clustered cases) in migrants from a single country from one of three geographic areas (eastern Europe, north Africa and sub-Saharan Africa). The lineage of the strains involved in the selected clusters was assigned based on the determination of lineage-specific SNP markers [15] by multiplex allele-specific oligonucleotide-PCR (ASO-PCR) [16].

Convenience samples from Valencia (all isolates with available WGS data in IBV, for the period 2004-2017) and Madrid (all isolates with genotypic data available in Hospital Gregorio Marañón, Spain) for the period 2004-10, were also included in the study. A retrospective convenience sample of part of the isolates from northern Morocco (Tangier, Tetouan and Larache) obtained during the same period also were included; no previous genotypic information was available for these isolates. Finally, a pool of 20 randomly selected TB migrant cases from Morocco (among all those diagnosed in Almería) that were infected with strains other than those analysed in this study were selected as controls.

### Genomic analysis

#### DNA purification

DNA for WGS of the MIRU-VNTR-defined clusters from Almería was purified from subcultures on Mycobacteria Growth Indicator Tube (MGIT) (using Qiagen kit; QIAamp DNA Mini Kit, Qiagen, Courtaboeuf, France) or Lowenstein Jensen medium (CTAB (cetyl trimethylammonium bromide)-based standard purification).

WGS of the strains from the collection in Morocco was performed by purifying (Qiagen kit) the DNA from the remnants of bacterial lysates that had been stored.

WGS of the strains from the collection in Madrid was performed by purifying DNA (Qiagen kit) from freshly inactivated suspensions from the stored frozen isolates.

#### Whole genome sequencing and single nucleotide polymorphism analysis

WGS was performed as detailed elsewhere [17]. Briefly, DNA libraries were generated following the Nextera XT Illumina protocol (Nextera XT Library Prep kit (FC-131–1024), Illumina, San Diego, United States (US)). Library quality and size distribution were checked on a 2200 TapeStation Bioanalyzer (Agilent Technologies, Santa Clara, US). Libraries were run in a Miseq device (Illumina), which generated 35–151–bp paired-end reads and an average per base coverage of 70 x. Sequences were deposited in www.ebi.ac.uk (PRJEB23664 and PRJEB25814).

We mapped the reads for each strain using the Burrows-Wheeler Aligner and the ancestral MTB genome, which was identical to H37Rv in terms of structure, but which included the maximum likelihood–inferred ancestral nt positions from a virtual ancestor [18]. SNP calls were made with SAMtools and VarScan (coverage of at least 20 x, mean SNP mapping quality of 20). From all the variants detected, we kept only the homozygous calls (those present in at least 90% of the reads in a specific position). Moreover, to filter out potential false positive SNPs due to mapping errors we omitted the variants detected in repetitive regions, phages and PE/PPE regions. Also, SNPs close to indels and those present in areas with an anomalous accumulation of variants (three or more SNPs in 10 bp) were omitted. Alignments and SNP variants (called with a > 20 x coverage in at least one of the isolates in a cluster) were visualised and checked for the remaining isolates in the Integrative Genomics Viewer IGV (version 2.3.59) programme. Multiple comparisons between the SNPs from different isolates were made using an in-house script written in R [19]. We used the reference values (in the number of SNPs) of Walker et al. [12] to determine whether the isolates in a MIRU-VNTR cluster were related. In three isolates we detected an unexpectedly high number of SNPs (> 200) with respect to the other members in the cluster; they were considered to be clustered as the result of homoplasy in the MIRU-VNTR pattern and therefore were eliminated from the study.

The median-joining networks were constructed from the SNP matrix generated for each case using the programme NETWORK 5.0.0.1. Median vectors (mv) were defined when the distribution of SNPs of the isolates analysed indicated the existence of a node that was not represented by the sampled isolates sequenced for each cluster. These median vectors therefore corresponded to non-sampled isolates in the cluster. The chronology of acquisition of SNPs is represented from left to right in the networks.

#### Cluster-specific single nucleotide polymorphisms and design of ASO-PCRs

To identify SNPs which were specific for cluster 113, we created a database of variants using sequences from isolates which were representative of the global MTB complex (MTBC) diversity. We downloaded all the accessible raw data from different publications [20-22]. All the fastq files published in these studies were downloaded and aligned against the ancestral MTB genome using the BWA tool. We kept the alignments that had a mean coverage higher than 20. Using this criterion, we kept 7,977 samples representative from the seven lineages. We extracted all the variants present in these samples as described above. The 7,977 samples were filtered to remove transmission clusters so we kept one representative strain of each transmission cluster detected. Once the transmission clusters were filtered, we kept 4,762 sequences. The 207,188 variants present in these samples were used to construct a reference database to evaluate the specificity of the SNPs selected for the ASO-PCRs to be applied in cluster 113.

Two different ASO-PCRs were designed to analyse strain 113. The first ASO-PCR aimed to differentiate new secondary transmitted cases in Almería from independently imported cases. We designed a four-plex single-tube format. Two of the four SNPs targeted were strain 113-marker-SNPs (one targeted the 113 allele and the other the non-113 allele). The remaining two SNPs targeted were only shared by the 113-strain isolates involved in the recent transmission cluster (Supplementary Table S1). The design pursued to obtain three different amplification patterns depending on whether a new case corresponded to recent transmission by strain 113, importation of strain 113 or infection with a strain other than 113.

The reaction conditions were as follows: 1.5 mM MgCl2, 0.2 μM of each primer (Supplementary Table S1), 200 μM deoxynucleotides (dNTPs) (Roche, Mannheim, Germany), 1% Dimethyl sulfoxide (DMSO) and 1.5 μL Taq DNA Polymerase (Roche, Mannheim, Germany). The PCR conditions were 95 °C for 5 min followed by 25–40 cycles (95 °C for 1 min, 61 °C for 1 min, and 72 °C for 1 min) and 72 °C for 10 min. The number of cycles was 25 when using as a template DNA purified from primary positive cultures and 40 when it was purified from sputa.

The second ASO-PCR was applied to assess whether an MTB isolate corresponded to strain 113 or to any other strain. We prepared another version of a four-plex single-tube ASO-PCR to target four SNPs (two alleles specific for isolates 113 and the other two alleles expected for non-113 strains) (Supplementary Table S2). Two different amplification patterns indicated whether a strain corresponded to the 113 strain or to any strain other than 113. The reaction conditions were as follows: 1.5 mM MgCl2, 0.2 μM of each primer (Supplementary Table S2), 200 μM dNTPs (Roche, Mannheim, Germany) and 1.5 μL Taq DNA Polymerase (Roche, Mannheim, Germany). The PCR conditions were 95 °C for 5 min followed by 30 cycles (95 °C for 1 min, 64 °C for 1 min, and 72 °C for 1 min) and 72 °C for 10 min. The ASO-PCR was applied on purified DNA purified or directly on bacterial lysates obtained from boiling stored frozen isolates.

The amplification patterns were analysed by sizing the amplification products using agarose gel electrophoresis.

## Results

**Figure 1:**
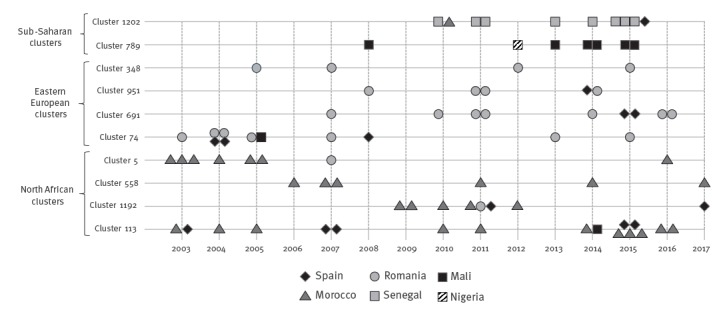
Chart summarising the general data of the clusters analysed, rich in cases from sub-Saharan Africa, eastern Europe and north Africa, 2003–2017 (n = 10 clusters)

We selected 10 MIRU-VNTR-defined clusters (Figure 1) from the universal molecular epidemiology survey that has been running in Almería since 2003. The clusters were rich in cases from countries representative of three wide geographic areas, namely, sub-Saharan Africa (two clusters, in which most cases were from Senegal and Mali), north Africa (four clusters in which most cases were from Morocco) and eastern Europe (four clusters in which most cases were from Romania). All the involved strains were pansusceptible and corresponded to lineage four.

Clusters are grouped according to their geographic origin (sub-Saharan, eastern European and north African). Each horizontal line corresponds to a cluster and each symbol corresponds to a patient. The patients involved in each cluster are distributed along the timeline (years at the bottom of the chart) with different symbols according to the nationalities shown in the legend.

### Sub-Saharan clusters

**Figure 2:**
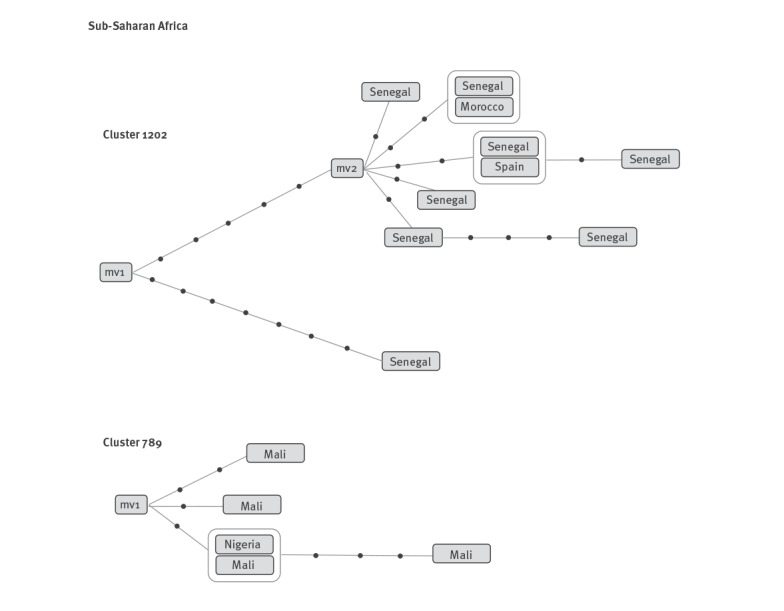
Networks of relationships obtained from the whole genome sequencing analysis for clusters rich in cases from sub-Saharan Africa

In cluster 1202, the analysis of SNPs from the 10 cases indicated the coexistence of a group of nine cases with a genetic distance of 0–7 SNPs between cases (Figure 2). The group included seven cases from Senegal, one from Morocco and one from Spain. Both observations strongly suggested that these nine cases were in fact part of a recent transmission event in Spain. Despite sharing an identical MIRU-VNTR pattern, the remaining case from Senegal showed a higher genetic distance i.e. 12 SNPs, with seven specific for this isolate and not sharing the five SNPs shared by all the isolates in the recent transmission group (Figure 2). These observations made it more likely, that this case corresponded to an unrelated importation from Senegal.

mv: median vectors; SNP: single nucleotide polymorphisms.

Each black dot corresponds to a SNP. Each box corresponds to a patient and the nationality is indicated within the box. When two or more cases share identical sequences (zero SNPs between them) they are surrounded by a line.

In cluster 789 (Figure 2), we sequenced five of the cases (four from Mali and the only case from Nigeria). The genetic distances between cases were 0–6 SNPs. No cases showed a distribution of SNPs that differed markedly within the group, suggesting the absence of independent importations from the country of origin.

### Eastern European clusters

**Figure 3:**
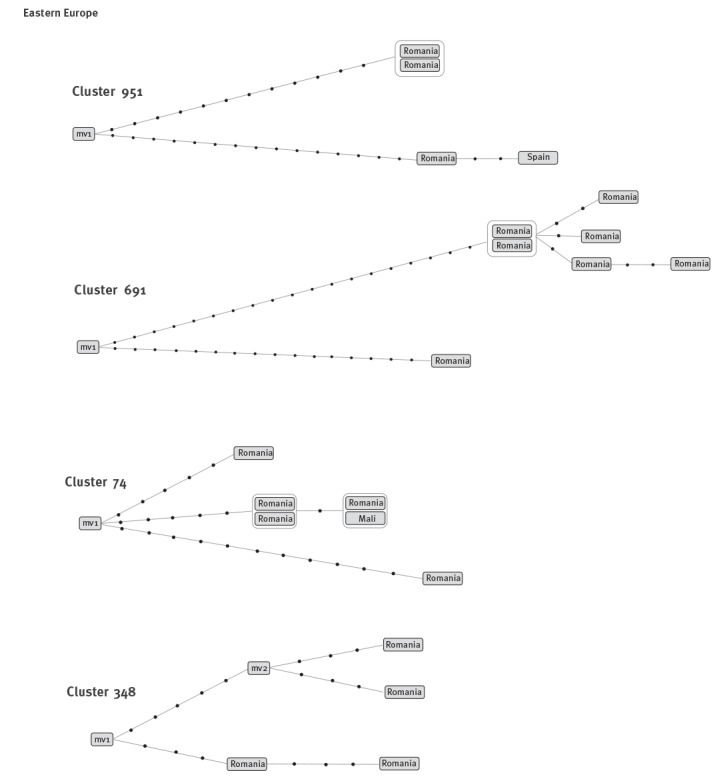
Networks of relationships obtained from the whole genome sequencing analysis for clusters rich in cases from eastern Europe

In three of the four clusters that were rich in cases from Romania (Figure 3), we detected the coincidence of cases due to either recent transmission or to independent importations.

mv: median vectors; SNP: single nucleotide polymorphisms.

Each black dot corresponds to a SNP. Each box corresponds to a patient and the nationality is indicated within the box. When two or more cases share identical sequences (zero SNPs between them) they are surrounded by a line.

In cluster 951, of the five cases, clustered by MIRU-VNTR, (Figure 3) WGS analysis of the four available isolates suggested that the theoretical cluster was hiding two independent subclusters. Two Romanian cases from the year 2011 differed in 27 SNPs and therefore corresponded to independent importations. Each case caused a secondary case in 2014 due to recent transmission in the host country. The isolates from the secondary cases had two SNPs (Spanish case) and zero SNPs (Romanian case) with respect to the corresponding index case.

A similar situation was observed for cluster 691 (Figure 3). WGS revealed that the MIRU-VNTR-defined cluster included two cases that brought together a high number of SNPs between them (35 SNPs), likely corresponding to two independent importations. A true recent transmission cluster had developed from one of these cases, with another five secondary cases occurring with genetic distances between cases of 0–5 SNPs. The other imported case corresponded to a dead-end branch i.e. it resulted in no secondary cases.

For cluster 74, we identified two different patterns (Figure 3). First, there were four highly related isolates, with 0–1 SNPs between cases, clearly indicative of recent transmission. Second, there were two branches, possibly corresponding to two independently imported cases with five and eleven specific SNPs, respectively, and did not share the five SNPs found in the four isolates belonging to the transmission subgroup. The transmission event (years 2003–2008) was caused by one of these likely imported cases, whereas the remaining two were representative of dead-end branches (years of isolation: 2013 and 2015).

Finally, in cluster 348 (Figure 3), two cases had a genetic distance of three SNPs, suggesting recent transmission between them. However, a definitive interpretation could not be found for the remaining two cases. The cases showed a genetic distance of six SNPs between them, but a non-sampled node (mv2) was inferred to be located between them in the network. It is, therefore, unclear whether these two cases are part of a recent transmission chain involving a non-sampled case in Spain or if they corresponded to two imported cases that were epidemiologically related with a non-sampled case at the host country.

### North African clusters

**Figure 4:**
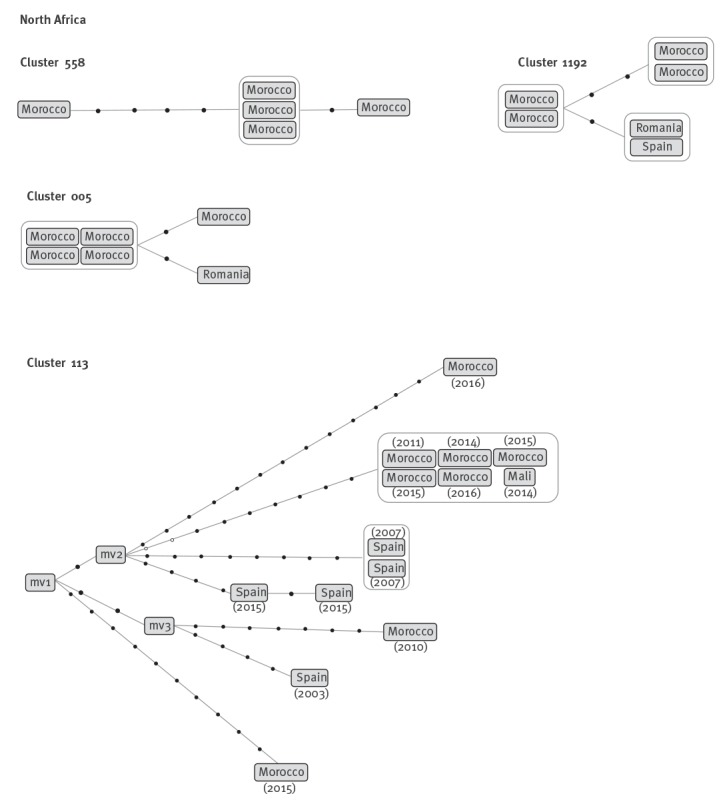
Networks of relationships obtained from the whole genome sequencing analysis for clusters rich in cases from North Africa

In three of the four clusters, predominately comprising of cases from Morocco, short genetic distances were recorded between all clustered cases (cluster 558: 0–5 SNPs, cluster 1192: 0–3 SNPs and cluster 5: 0–2 SNPs between cases), highly indicative of recent transmission in the host country, Spain (Figure 4).

mv: median vectors. SNP: single nucleotide polymorphisms.

Each box corresponds to a patient and the nationality is indicated within the box. When two or more cases share identical sequences (0 SNPs between them) they are surrounded by a line. Each black dot corresponds to a SNP. White dots detailed in cluster 113 correspond to non-fixed alleles, found in heterozygosis in one of the cases, but in homozygosis in the remaining cases. The years of diagnosis are indicated in brackets.

However, for the remaining cluster, cluster 113, which included 17 cases, WGS of the 14 available isolates revealed a much more complex network of relationships (Figure 4).

Three median vectors (mv) corresponding to non-sampled cases had to be defined. Seven independent branches were observed (Figure 4), with four, four, seven, eight, nine, 10 and 13 specific SNPs for each of the branches and each more likely corresponding to unrelated cases (distances between each two branches were in the range of 11–24 SNPs). Therefore, these cases were likely due to unrelated importations from Morocco. Of the seven branches, four corresponded to dead-ends, including a single case each (years 2003, 2010, 2015, and 2016); three were from Morocco and were diagnosed 10, 6, and 2 years after arrival. As there were no additional related secondary cases, the findings seem consistent with likely reactivations.

Two of the remaining three branches showed one additional case that was closely related to the imported index case in each branch (zero and one SNPs), which was diagnosed the same year as the index case (year 2007 and 2015, respectively), possibly due to self-limited recent transmission events in Spain.

The remaining branch was the only one with a higher number of cases i.e. six, among which no SNPs were found. Of note, two alleles were in heterozygosis in one of these cases (year 2011) and were fixed as homozygotes in the remaining five cases. Based on this observation, we can infer that the case with heterozygosis was the index case and the remaining five cases were secondary cases and likely due to recent transmissions in Spain.

### New strategy based on whole genome sequencing data to precisely identify recent transmission

In our context, MIRU-VNTR was proved useless, because it could not discriminate between the three events observed for strain 113 e.g. dead end-imported hosts, self-limited transmission chains and ongoing active transmission events. Among the 17 cases theoretically linked by MIRU-VNTR, only six were really involved in an active recent transmission chain whereas the remaining 11 cases had been misclassified and their epidemiological follow-up was not well oriented. Using standardised interviews with the cases it was possible to establish epidemiological links between the cases in the six-case subcluster, revealing that three cases were customers of the same bar and another case shared a flat with them.

In order to be able to precisely identify the true secondary cases in an active transmission chain, we defined a new approach. We first identified the 71 common SNPs shared by all members in MIRU-VNTR-defined cluster 113 and those SNPs which were specific for the different branches in the network. We designed an allele-specific multiplex PCR (ASO-PCR) including four PCRs, which targeted the following (Supplementary Table S1): (i) two SNPs specific for all the strain 113 isolates in the network, which were selected as a general marker for this strain (one PCR targeting the 113 allele in one of these SNPs and the other PCR the non-113 allele from the other SNP) and (ii) two SNPs among the nine SNPs that were only shared by the branch including the active transmission subcluster (targeting the alleles for the active transmission subcluster).

**Figure 5:**
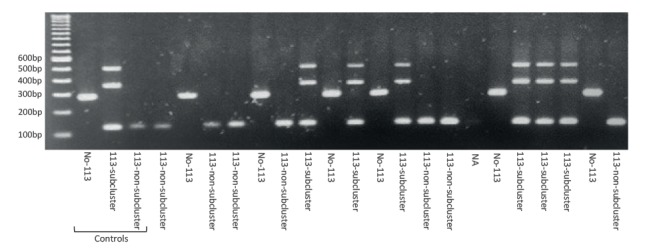
Results for the multiplex ASO-PCR designed to precisely assign new incident cases infected by the strain 113 in Almería and labelling them as due to recent transmission or importation.

The ASO-PCR was designed following a four-plex format to target the four SNPs simultaneously in the same reaction tube. This lead to three different amplification patterns depending on whether a new case corresponded to the recently transmitted subcluster 113, to a 113 isolate not involved in this active subcluster (therefore corresponding to a new unrelated importation) or to a strain other than 113 (Figure 5). The specificity of the multiplex ASO-PCR was checked by testing all the 14 isolates with the 113 VNTR pattern and a selection of 20 randomly selected strains for Moroccan migrants among those diagnosed in Almería. The expected pattern for the three possible profiles was obtained in all cases.

Amplification patterns obtained from a selection of isolates representative of the 113 transmission subcluster (113 subcluster), isolates 113 not included in the recent transmission subcluster (113 non-subcluster) and isolates other than 113 (No 113). The different amplification patterns for each group can be observed.

The PCR was transferred to Torrecardenas Hospital in Almería to be prospectively applied on all newly diagnosed TB cases of Moroccan origin or living in the same area as the cases involved in the MIRU-VNTR-defined cluster 113. We first checked that the PCR was sensitive enough to be applied directly on respiratory specimens and were able to obtain an interpretable profile when decontaminated sputa with high or medium bacillary load were used as templates.

**Figure 6:**
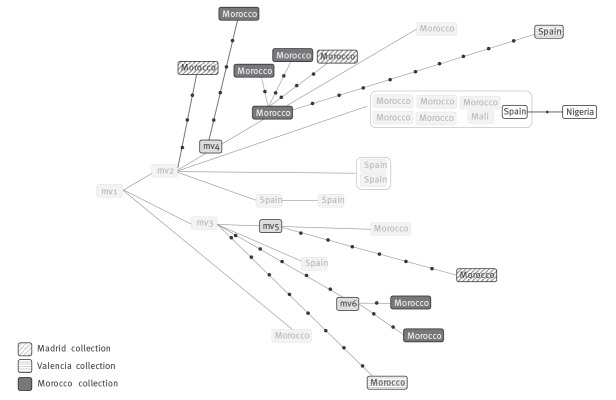
Extended network of relationships obtained from the whole genome sequencing analysis for cluster 113 including Almería, Madrid, Valencia and Morocco isolates

An interpretable result was obtained for all the eight stain-positive cases in which the multiplex ASO-PCR was prospectively applied (during a 3-month period) directly on sputa. For the prospective cases with paucibacillari sputa it was necessary to wait until culture was available. In 15 cases, the pattern corresponded to a non-113 strain; however, in two cases (one from Spain in 2016 and the other from Nigeria in 2017), we obtained the pattern expected for active subcluster 113. Both isolates shared the expected 113 MIRU-VNTR pattern. Subsequent WGS analysis indicated that they showed zero and one SNPs with the six isolates previously included in the active subcluster (Figure 6).

mv: median vectors; SNP: single nucleotide polymorphisms.

Each box corresponds to a patient and the nationality is indicated within the box. When two or more cases share identical sequences (0 SNPs between them) they are surrounded by a line. Each black dot corresponds to a SNP. Isolates in Almería detailed in Figure 4 are now fainted. The two new cases identified in Almería by applying the ASO-PCR are shown in white boxes.

### Expanded analysis of strain 113 in unrelated populations

Once the demand for identification of new cases due to recent transmission of the active transmission node was resolved, we focused on the other issue affecting this cluster i.e. the independent importations of closely related (genetically) strains from the country of origin, those likely prevalent in Morocco that have acquired diversity by circulating over extended periods of time. We tried to identify other examples of independent importations for this strain in other unrelated populations.

For this purpose, we selected two Spanish populations: one from Valencia (eastern Spain), a representative of a node with WGS data available from a population-based genomic epidemiology programme and another one from Madrid (central Spain), for which no population-based WGS data were available.

The approach in Valencia was direct and limited to querying on the presence of the 71 SNPs that are specific for the isolates in cluster 113; we identified two cases sharing all the 71 SNPs. When these were integrated into the Almería network, they consistently corresponded to two new subbranches in two of the previously described importation branches (Figure 6).

**Figure 7:**
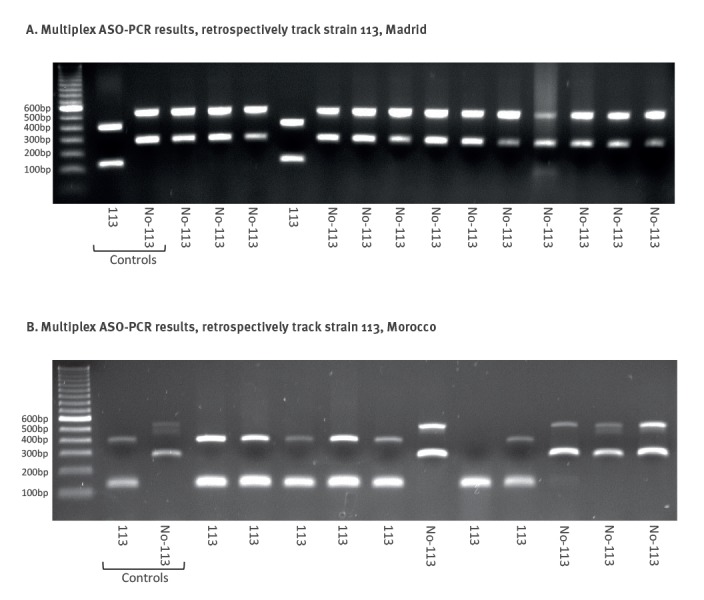
Results for the multiplex ASO-PCR designed to (A) retrospectively track strain 113 in Madrid and (B) retrospectively track strain 113 in Morocco

The approach in Madrid was indirect, involving application of a multiplex ASO-PCR directly on stored isolates from Moroccan migrant TB cases. We prepared a new version of a four-plex ASO-PCR to target four SNPs. Two of the PCRs targeted the alleles that were specific for isolates 113 and the other two targeted the alleles expected for non-113 strains (Supplementary Table S2); the two amplification patterns identified indicated whether a strain corresponded to the 113 MIRU-VNTR cluster or to any strain other than 113 (Figure 7). We applied it to 134 available Moroccan isolates from our retrospective convenience sample and detected the 113 pattern in five cases (Figure 7a). WGS of three of these isolates confirmed them to be 113 (they included all 71 SNPs) and their integration in the network revealed three new branches (Figure 6).

The different amplification patterns for the strains belonging to the 113 group and those that do not are observed.

### Expanded analysis of strain 113 in the country of origin

We completed the general analysis of strain 113, with a cross-border analysis, by tracking its circulation in the country of origin. The epidemiological information collected from cases by interview aided in determining that most migrant cases were from cities in the north of Morocco.

Molecular epidemiology studies in northern Morocco were checked in which MIRU-VNTR genotypes corresponding to strain 113 could be found. Chaoui et al. [23] reported a cluster involving four cases in Tangier infected by a LAM3 SIT33 strain that could correspond to strain 113. However, only data for the 12-loci version of MIRU-VNTR were available.

To confirm whether strain 113 was circulating in the area, as suggested by the published data, the same multiplex ASO-PCR that had been designed to track strain 113 in Madrid was transferred and locally applied in Morocco. Interrogation of 11 SIT33 isolates revealed seven with the pattern corresponding to strain 113. In addition, testing of 45 additional retrospective isolates from northern Morocco (Tangier, Tetouan and Larache), for which no previous genotypic information was available, revealed a 113 pattern in seven isolates (Figure 7b). WGS was performed in six of the 14 isolates that were positive for 113 and enabled us to integrate them into the network of relationships (Figure 6). Three of the isolates were positioned in two new sub-branches and the other three were located in one of a previously defined importation branch. Furthermore, two probable recent transmission events in Morocco, involving two and three cases respectively, were identified indirectly (with three SNPs between cases in both of them).

## Discussion

Molecular epidemiology based on universal genotyping of TB cases in a population allows us to identify clustered cases that are infected by *M. tuberculosis* isolates with identical fingerprints. From the analysis of clustered cases, we can obtain valuable data on transmission dynamics in different epidemiological scenarios.

The increased complexity resulting from changing socio-epidemiological features due to migration demands special attention. The clusters may be autochtonous, mixed multinational, and foreign-born clusters rich in cases from a specific country.

Some of the complex molecular clusters identified in populations with a higher percentage of migrants are not always accompanied by clear epidemiological links between the cases involved [7,24,25]. Here, we tried to analyse whether the lack of epidemiological support could mean that some of the clusters involving migrants were not robust and were misleadingly alerting us to recent transmissions.

We hypothesised that some of the cases in these clusters could correspond to independent importations of strains that might be prevalent in the country of origin. Genetic diversity would be expected to accumulate for a prevalent strain circulating in a high-incidence country over extended periods of time. However, the diversity accumulated is probably insufficient to lead to a change in the MIRU-VNTR pattern, thus explaining why unrelated cases independently importing these strains appear clustered. MIRU-VNTR types are conserved for highly prevalent strains, as reported in Denmark for a highly prevalent strain responsible for 35% cases over 15 years [26]. However, the application of more discriminative methods e.g. WGS, could help us to reveal some degree of diversity between these prevalent strains and differentiate between true recent transmission in the host country (when no or very limited genetic diversity is found between the corresponding isolates) and independent, unrelated importations of prevalent strains in the country of origin (if we detect greater genetic distance).

Application of this strategy, following the consensus thresholds of diversity to assign or rule out recent transmission with WGS [12], revealed that unrelated importations were hidden within some MIRU-VNTR-defined clusters and had been misinterpreted as recent transmissions in the host country. Due to the size of certain clusters in the analysis we only revealed a minority (one case in several clusters) that had been misclassified as recent transmission when it was really due to importation. However, in some of the bigger clusters, the magnitude of misclassified cases revealed was higher (eight of 14).

In a 2016 publication, Stucki et al. [27] reported importations within MIRU-VNTR clusters in a nationwide analysis in Switzerland (90 patients in 35 clusters during 2000–08). Only 25% of the MIRU-VNTR-defined clusters including migrants (in this case, mostly from east Africa) were refuted using WGS. The clustering proportion fell from 16.7% to 6.5% for migrant clusters; when only Swiss-born clusters were considered, the decrease was smaller (19.3% to 14.3%). In addition, descriptions of misassignation of recent transmission in MIRU-VNTR-proven migrant clusters revealed by WGS have recently been reported in Canada [28] and the Netherlands [29].

Although our findings are limited to the low number of clusters selected, both these data and ours suggest that the involvement of genetically closely related strains imported independently from high-incidence regions is a widespread phenomenon. We cannot extend the findings from the migrant clusters in our study to all clusters including migrants because in our setting some migrant nationalities were not represented. Nevertheless, our results showed that this phenomenon was not anecdotal or restricted to specific geographic areas and that it was found in clusters with migrants that were representative of different areas e.g. eastern Europe, north Africa and sub-Saharan Africa.

In our study, the identification of imported cases within clusters defined by standard genotyping was mainly supported by the analysis of the total number of SNPs between the clustered cases. However, the analysis of the chronology of diagnosis of the TB cases can also be useful to identify importations. This is because the order of emergence of SNPs is sequential and once acquired they do not reverse [30]. In cluster 558, the last case diagnosed (year 2014) did not present the four SNPs identified in the remaining clustered cases, diagnosed 3–8 years earlier. The most likely explanation is that the 2014 case was imported from a more ancestral branch than the one involved in the recent transmission event in Spain.

The demonstration that both importations and recent transmissions could co-exist in a cluster defined by standard genotyping raised an alert: once one of these genetically closely related strains is imported into a host country, standard molecular epidemiology–surveillance approaches are of very limited value. Based on standard MIRU-VNTR, it would be impossible to discriminate between secondary cases that originated in the host country and unrelated independent importations: all cases would be equally considered clustered.

It is important to differentiate between a new imported case and a recently transmitted secondary case, because each represents a completely different epidemiological situation that has to be managed separately. Consequently, other authors have recommended WGS as the only way to ensure more accurate identification of recent transmission, particularly among migrants from high-incidence areas [27,31]. An alternative to the analysis based on WGS and SNPs calling based on pipelines is the technique of core genome MLST typing, which takes advantage to the discriminatory power of the next generation sequencing (NGS) technique and makes easier the SNP calling by standardised processing and allows a more direct comparative analysis across different laboratories [32]. However, global implementation of WGS is expensive and WGS has been successfully implemented at population level in few settings only [33-35]. With the aim to overcome these limitations and to find a solution that can be implemented in settings where nationwide WGS application is not a reality, we adapted a strategy previously developed by our group to survey high-risk strains. This strategy is based on tailored ASO-PCRs targeting strain-specific SNPs identified from WGS data of representative isolates for the strains to be surveyed [36]. We implemented it in previous studies to be able to provide a fast response to challenges, such as optimising surveillance of transmission of actively transmitted strains [36], rapid tracking of the presence of specific outbreak strains in a population [37] and confirming the presence of secondary cases due to imported XDR strains from Russia directly on respiratory specimens in the hospital setting [38]. In the current study we adapted the strategy to tailor PCRs targeting the SNPs that were specific for isolates actively involved in recent transmission in the host country and to differentiate these isolates from other independently imported isolates which lacked those SNPs.

To pilot this strategy, we selected the most complex cluster in our study, namely cluster 113, which was rich in cases from Morocco (six different importation branches together with an active transmission cluster). The strategy prospectively identified new secondary cases directly from respiratory specimens. Our proposal not only resolved the epidemiological challenge at the local level, but also enabled us to expand the boundaries of our analysis to other unrelated populations in Spain. If this strain corresponded to a prevalent strain in the country of origin, we would be able to find it in unrelated populations receiving migrants from Morocco. We identified the strain in the two unrelated populations surveyed and proved that importations of the same strain occurred in other settings, thus showing that they were not the result of recent transmissions. For some of the remaining studied strains from migrants from Morocco we also found data indicating they are circulating also in Morocco [23,39] and similar efforts could be done to fully characterise their global distribution.

## Conclusion

Tracking transmission of TB through cross-border surveillance is a crucial element in the current epidemiological surveillance of TB, and data from both the country of origin and host countries must be integrated as recently exemplified in a study revealing a cross-border MDR-TB cluster involving several European countries [40]. Our findings revealed standard MIRU-VNTR-based epidemiology was not a suitable approach for cross-border surveillance as it was unable to discriminate between importations and recent transmissions. WGS-based analysis was able to differentiate these two overlapping events, however, genomic analysis is not accessible for many countries involved in cross-border TB transmission. Here, we propose a new strategy, adapted to settings with no or limited access to WGS , based on designing simple PCR tools tailored to be adapted to identify either recent transmission in the host country or independent importations from the country of origin. Adapted versions of the same PCRs were also designed to be transferred and applied to track the strain circulating in the country of origin.

Our next step will be to extend the approaches used in this study to develop a network of nodes surveying prevalent strains from countries with a high TB incidence that are being exported to countries with low-TB burden. Such a network could contribute to the establishment of a new global cross-border surveillance system, fitted to the challenges associated with international migration.

## Supplementary Data

353000 Supplementary Tables
