# Supplementary material for: Whole genome sequencing–based analysis of tuberculosis (TB) in migrants: rapid tools for cross-border surveillance and to distinguish between recent transmission in the host country and new importations
Source: Euro Surveill. 2019 Jan 24;24(4):1800005. doi: 10.2807/1560-7917.ES.2019.24.4.1800005 (PMC6351995; doi:10.2807/1560-7917.ES.2019.24.4.1800005)
Supplement: Supplementary Tables [file 1800005_GARCIAdeVIEDMA_SupplementaryTables.pdf]

This supplementary material is hosted by Eurosurveillance as supporting information alongside the article “Whole -genome sequencing–based analysis of tuberculosis (TB) in migrants: rapid tools for cross-border surveillance and to distinguish between recent transmission in the host country and new importations” on behalf of the authors who remain responsible for the accuracy and appropriateness of the content. The same standards for ethics, copyright, attributions and permissions as for the article apply. Eurosurveillance is not responsible for the maintenance of any links or email addresses provided therein.

Supplementary Table S1. ASO- PCR designed to differentiate between new secondary transmitted cases in Almería from independently imported cases.

SNP	Gene	SNP location	PCR Fragment size	DNA target	Primer sequences
SNP1	Rv3276c	G 3659433 A	465 bp	Almería transmission 113 strain	Rv3276c-R-mut: 5'-GCGCGGGGGTTACGA**T**-3'
					Rv3276c-F: 5'-AGCGGGTAGTCCAAGACC-3'
SNP2	Rv1028c	C 1151418 T	333 bp	Almería transmission 113 strain	Rv1028c-F-mut: 5'-GGGAAACCTGGCACCC**T**-3'
					Rv1028c-R: 5'-AGATAGCCCAGCAGGATGG-3'
SNP3	Rv3915	G 4403614 A	259 bp	NO 113 strain	Rv3915-R-wt: 5'-GCAAGTCCGTACTCACG**C**-3'
					Rv3915-F: 5'-GTGCCTTCCAACAGCATCG-3'
SNP4	Rv1236	G 1379613 C	119 bp	113 strain	Rv1236-R-mut: 5'-GTCCAGGGTCCTGAAGAG**G**-3'
					Rv1236-F: 5'-AGGATCTGCTAAGAGCAGCG-3'

Supplementary Table S2. ASO- PCR designed to assess whether an MTB isolate corresponded to strain 113 or to any other strain.

SNP	Gene	SNP location	PCR Fragment size	DNA target	Primer sequences
SNP5	Rv0462	C 553675 T	495 bp	NO 113 strain	Rv0462-F-wt: 5'-CGACGTTCTGTCAGCCAAA**C**-3'
					Rv0462-R: 5'-GCGACTCCATTGACTCACC-3'
SNP6	Rv3131	G 3496697 A	374 bp	113 strain	Rv3131-F: 5'-GGCGTCAAGTGTCGTTAGG-3'
					Rv3131-R-mut: 5'-CCGGATCGGTGCTACG**T**-3'
SNP3	Rv3915	G 4403614 A	259 bp	NO 113 strain	Rv3915-R-wt: 5'-GCAAGTCCGTACTCACG**C**-3'
					Rv3915-F: 5'-GTGCCTTCCAACAGCATCG-3'
SNP4	Rv1236	G 1379613 C	119 bp	113 strain	Rv1236-R-mut: 5'-GTCCAGGGTCCTGAAGAG**G**-3'
					Rv1236-F: 5'-AGGATCTGCTAAGAGCAGCG-3'
